# Optimization of Layered Cathode Materials for Lithium-Ion Batteries

**DOI:** 10.3390/ma9070595

**Published:** 2016-07-19

**Authors:** Christian Julien, Alain Mauger, Karim Zaghib, Henri Groult

**Affiliations:** 1 Physicochimie des Electrolytes et Nanosystèmes Interfaciaux (PHENIX), Sorbonne Universités, UPMC Univ. Paris 06, CNRS UMR 8234, 4 place Jussieu, Paris 75005, France; henri.groult@upmc.fr; 2 Institut de Minéralogie, de Physique des Matériaux et de Cosmochimie (IMPMC), Sorbonne Universités, UPMC Univ. Paris 06, CNRS UMR 7590, 4 place Jussieu, Paris 75005, France; alain.mauger@impmc.jussieu.fr; 3 Institut de Recherche d’Hydro-Québec (IREQ), Stockage and Conversion d’Energie, 1800 Lionel-Boulet, Varennes, QC J3X 1S1, Canada; zaghib.karim@ireq.ca

**Keywords:** nanomaterials, layered compounds, cathodes, lithium-ion batteries

## Abstract

This review presents a survey of the literature on recent progress in lithium-ion batteries, with the active sub-micron-sized particles of the positive electrode chosen in the family of lamellar compounds Li*M*O_2_, where *M* stands for a mixture of Ni, Mn, Co elements, and in the family of *y*Li_2_MnO_3_•(1 − *y*)LiNi_½_Mn_½_O_2_ layered-layered integrated materials. The structural, physical, and chemical properties of these cathode elements are reported and discussed as a function of all the synthesis parameters, which include the choice of the precursors and of the chelating agent, and as a function of the relative concentrations of the *M* cations and composition *y*. Their electrochemical properties are also reported and discussed to determine the optimum compositions in order to obtain the best electrochemical performance while maintaining the structural integrity of the electrode lattice during cycling.

## 1. Introduction

**Figure 1:**
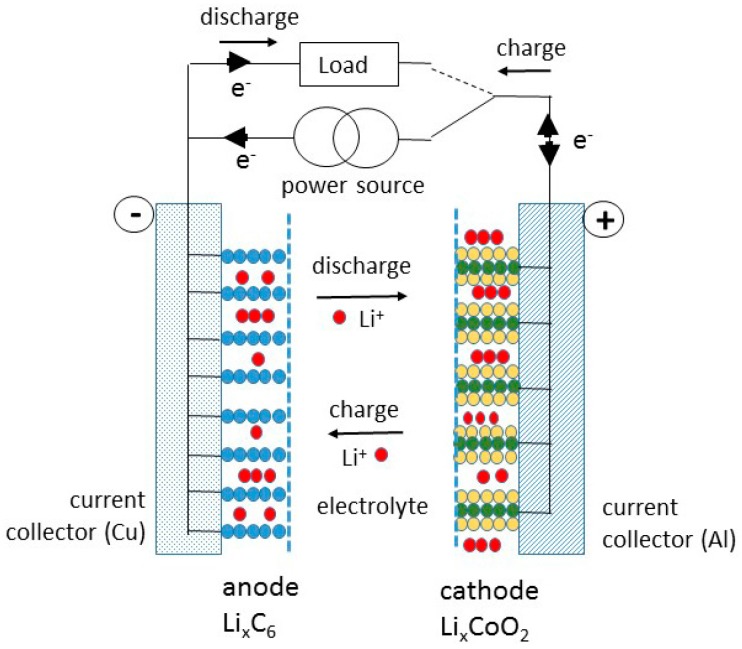
Schematic figure of a Li-ion battery. LiCoO_2_ is used as a cathode and graphite as an anode.

Lithium-ion batteries (LiBs) are generally composed of two electrode compounds having an open structure, which act as host frameworks for the insertion/de-insertion of Li^+^ ions and are the place of charge transfer. The LiB prototype was composed of graphite as a negative electrode (often named anode) and a transition-metal oxide (TMO), i.e., LiCoO_2_, LiMn_2_O_4_, etc., as a positive electrode (often named cathode), separated by the electrolyte that provides a transport medium for ions (Figure 1). During the charge of the LiB charges, the Li^+^ ions are extracted from the cathode and inserted into the anode, while the electrons pass through the outer circuit (load). Consequently, the effectiveness of a lithium-ion cell is dependent on the availability of crystallographic sites for hosting Li^+^ ions, in other words on the insertion mechanism and thereby on the transport properties of ions and electrons in both electrode materials. Note that in the case of LiB with the graphite/TMO configuration, the limiting factor comes from the cathode side [1], in which the redox reaction is described by:

(1) $$LiM_{a}^{n}O_{b}↔Li_{1-x}M_{a}^{n+x}O_{b}+xLi^{+}+xe^{-}$$

As the transition metal entering the composition of the active element of the cathode is oxidized and reduced during the cell charge and discharge, respectively (Equation (1)), the cathode is primarily involved in the cathode process and then in the electrochemical performance of the cell, i.e., potential, specific capacity, energy density, rate capability, etc. [2,3].

Conventional rechargeable Li batteries exhibit rather poor rate performance, even compared with old technologies such as lead-acid [4]. Achieving high rate rechargeable Li-ion batteries depends ultimately on the dimension of the active particles for both negative and positive electrodes [5]. One of the prospective solutions for the preparation of electrodes with high power density is the choice of nanocomposite materials because the geometric design of the insertion compound is a crucial intrinsic property from the viewpoint of structural stability and low kinetics of ions in oxide. 

The theoretical capacity of a given electrode material, which influences the C-rate estimations, is calculated by the following equation:

(2) $$Q_{\mathrm{th}}=\frac{e\times N_{A}}{M_{w}\times 3.6}$$

where *Q*_th_ is the theoretical specific capacity (mAh·g^−1^), *M*_w_ is the mass of the correlated component (g·mol^−1^), and *Z*, *N*_A_, and *e* represent the number of electrons involved in the reaction, Avogadro’s number, and electronic charge, respectively. As an example, let us consider the theoretical capacity of the Li-rich layered material Li_1.2_Ni_0.2_Mn_0.6_O_2_ or 0.5(Li_2_MnO_3_)•0.5(LiNi_0.5_Mn_0.5_O_2_). *Q*_th_ of Li_2_MnO_3_ and LiNi_0.5_Mn_0.5_O_2_ are 458 mAh·g^−1^ and 273 mAh·g^−1^, respectively. Taking into account the molar concentration of the compounds in the Li-rich electrode, the overall theoretical capacity is expected to be 378 mAh·g^−1^.

The performance of electrode materials for Li-ion batteries reached today is the result of intensive research to reduce the size of particles to the nanoscale for three main reasons. One is the increase of the effective contact area of the powder with the electrolyte. A larger effective contact surface with the electrolyte means a greater probability to drain Li^+^ ions from the electrode, which increases the power density of the cell. Secondly, nano-sized particles have larger surface areas and exhibit superior charge transfer kinetics. Thirdly, a smaller particle size also reduces the diffusion pathway of Li^+^ ions to the interior of the particle, which leads to a greater capacity at higher charge/discharge rates and therefore to a larger power density [6,7]. Formally, the characteristic time (or Li^+^ migration time), *τ*, for the intercalation reaction is deduced from Fick’s law:

*τ* = *L*^2^/4πD* (3)

where *L* is the diffusion length and D* the chemical diffusion coefficient of Li^+^ ions in the host lattice [7]. For a given chemical diffusion coefficient of Li^+^ ions, D*, the reduction of the size of the active particles from micro- to nano-scale implies a decrease in the characteristic time *τ* for the intercalation reaction by a factor of 10^6^, which corresponds to an enhancement of rate capability of the electrode. Nanoparticles, as well as more tailored nanostructures, are being explored and exploited to enhance the rate capability, even for materials with poor intrinsic electronic conductivity such as olivine frameworks. Therefore, in the present work, only materials under the form of nano-sized particles are investigated.

**Figure 2:**
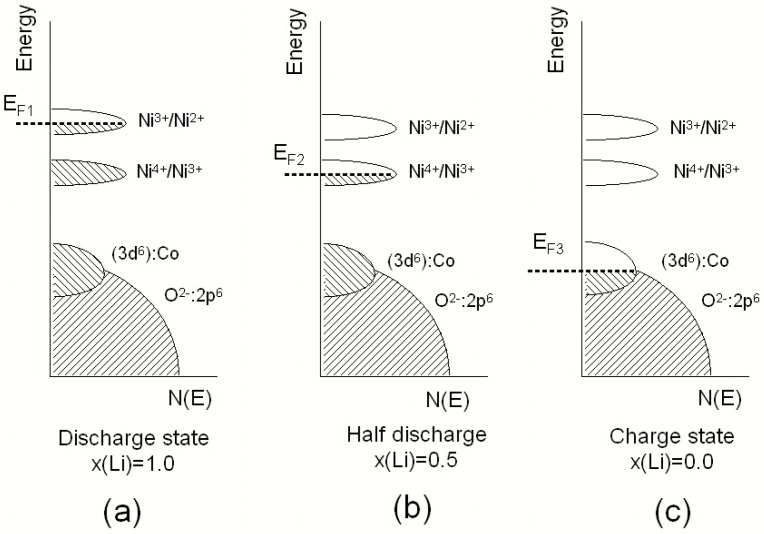
Schematic representation of the energy diagram vs. density of states for Li_x_Ni_1/3_Mn_1/3_Co_1/3_O_2_ at different charge states. The Fermi level of the cathode material is represented for three state of charge. (**a**) Full discharge; (**b**) half discharge; (**c**) full charge.

Among the materials capable of delivering high reversible capacity, the layered rhombohedral structures ($R\bar{3}m$ space group) that are part of the solid-solution series Li(Ni_y_Mn_z_Co_1-y-z_)O_2_ (called NMC hereafter) were first introduced by Liu et al. [8]. The symmetric LiNi_1/3_Mn_1/3_Co_1/3_O_2_ compound was proposed by Ohzuku’s group in 2001 [9]. These materials are now widely studied as alternative 4-volt cathode materials to replace LiCoO_2_, exhibiting much higher voltage, great structural stability, and enhanced safety even at elevated temperature and higher reversible charge capacity [10,11,12]. However, the electrochemical performance of NMCs, i.e., capacity retention and long life cycling, strongly depend on the composition, the particle morphology, and the deviation from the ideal rock-salt structure [13]. The reversible specific capacity of NMC was measured to be 160 mAh·g^−1^ in the cut-off potential range of 2.5–4.4 V and 200 mAh·g^−1^ in that of 2.8–4.6 V [14]. In the Li_1−x_Ni_1/3_Mn_1/3_Co_1/3_O_2_ cathode, the charge/discharge process occurs with different oxidation states: the Ni^2+^/Ni^4+^ in the range 0 ≤ *x* ≤ 2/3 and the couple Co^3+^/Co^4+^ is activated in the range 2/3 ≤ *x* ≤ 1, while the electrochemically inactive Mn^4+^ ions play an important role by stabilizing the electrode structure [15]. The schematic representation of the energy diagram vs. density of states for Li_x_Ni_1/3_Mn_1/3_Co_1/3_O_2_ is shown in Figure 2 for three states of charge (SOC). During the charge process, the Fermi level of the host material, *E*_F_, is lowered and for a high degree of delithiation *E*_F_ is pinned at the top of the O(2p) band, which provides an intrinsic voltage limit for the cathode material [16].

**Figure 3:**
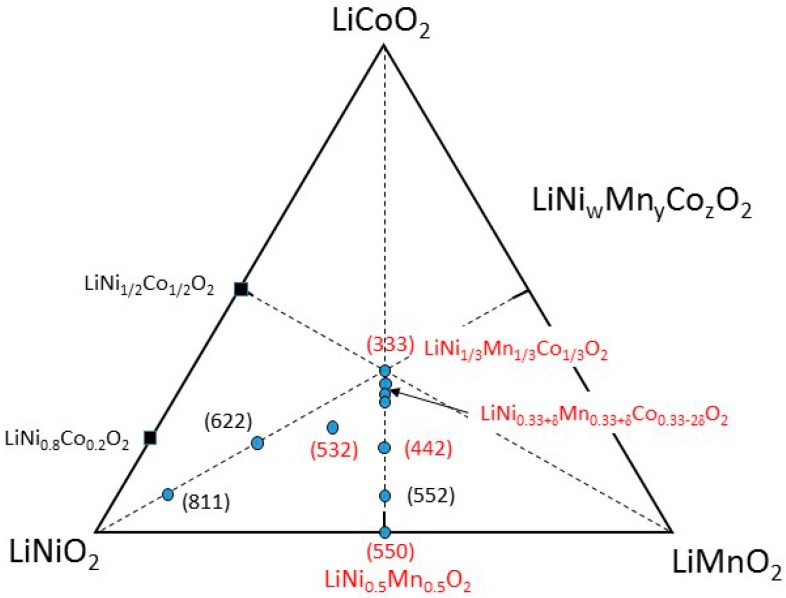
Ternary phase diagram of the mixed transition-metal oxides LiNi_w_Mn_y_Co_z_O_2_ (*w* + *y* + *z* = 1) formed by the LiCoO_2_-LiNiO_2_-LiMnO_2_ solid solutions. The compounds studied in this work are marked in red. The samples are noted (*w’y’z’*) where *w’* = 10*w*, *y’ =* 10*y* and *z’ =* 10*z*.

In this work, nanostructured NMC cathode materials with different chemical configurations (see ternary phase diagram, Figure 3 were synthesized by wet-chemical methods. As the electrochemical performance of NMC materials is extremely dependent on the synthesis method and parameters, we report the influence of the recipe, the particle size and morphology, and the sample composition on electrochemical properties of NMC electrode materials. In addition to the cation mixing (structural defect), the crystallinity, phase purity, particle morphology, grain size, and surface area depend on the synthesis method, and they all play an important role in ionic and electronic transport [17]. A series of mixed transition-metal oxides LiNi_w_Mn_y_Co_z_O_2_ samples is investigated for which the following parameters affecting their structural and electrochemical properties are considered: (i) effect of particle size; (ii) effect of the cation mixing; (iii) adjustment of the transition-metal/lithium ratio of the precursor; (iv) effect of chelating agent used in the synthesis; (v) effect of the synthesis recipe; (vi) slight deviation of the cobalt content in the symmetric NMC compound; and (vii) Li-rich integration in layered powders.

## 2. Experimental

### 2.1. Synthesis Procedures

Layered compounds were synthesized by either solid-state reaction or wet chemistry (“chimie douce”). These solution methods consist of acidification of aqueous solution of the starting compounds was used to prepare the layered cathode materials. The acidification is generally realized by using carboxylic acids. Three classes can be considered according the synthetic process: sol-gel, co-precipitation, and combustion [18,19,20]. The synthesis routes for the different samples are as follows: (1) LiNi_0.55_Co_0.45_O_2_ and NMC powders were prepared by solid-state reaction at 850 °C in air as shown elsewhere [21]; (2) LiNi_0.55_Co_0.45_O_2_ nano-powders were synthesized by hydrothermal method using acetate raw materials [22]; (3) a series of mixed transition-metal oxides LiNi_w_Mn_y_Co_z_O_2_ (*w* + *y* + *z* = 1) were synthesized by the co-precipitation method. This consists of a hydroxide route using transition-metal hydroxide and lithium carbonate as starting materials, as reported elsewhere [23]. (4) Li_1+x_(Ni_1/3_Mn_1/3_Co_1/3_)_1-x_O_2_ powders were prepared by a hydroxide route using an aqueous solution in which the pH was controlled with care using simultaneously NaOH and NH_4_OH fed into the reactor; (5) a series of symmetric LiNi_1/3_Mn_1/3_Co_1/3_O_2_ powders was prepared by the precipitation technique using metal acetates as raw materials and succinic acid as a chelating agent. Different acid to metal-ion molar ratios *R* were used to study the effect of this parameter on the structural properties of the final product; (6) A series of NMC powders were synthesized by co-precipitation assisted by single dicarboxyl (oxalic) and complexed dicarboxyl (tartaric) acid; (7) a series of LiNi_0.33+*δ*_Mn_0.33+*δ*_Co_0.33-2*δ*_O_2_ with different values of *δ* was obtained by the sol-gel route assisted by citric acid (tricarboxyl) in keeping the pH of the solution in the range 5–6; (8) Li-rich Li(Li_1/3-2x/3_Ni_x_Mn_2/3-x/3_)O_2_ (0 ≤ *x* ≤ 0.5) powders were synthesized by a citrate–gel method using acetate salts. Citric acid was dropped wisely to the solution under continuous stirring for 6 h with molar ratio 1:1 of (Li + Ni + Mn):C_6_H_8_O_7_ to adjust the pH value to 7–8 with ammonium hydroxide. All final products were obtained by sintering the powders at an optimum sintering temperature of *T* = 900 °C in air for a few hours.

### 2.2. Characterizations

The structure of the samples was investigated using X-ray diffractometer (XRD) (PANalytical X’Pert, Lelyweg, The Netherlands) using nickel-filtered Cu-K*α* radiation. The diffractograms were taken at room temperature in the 2*θ* range 10°–80°. Thermogravimetry (TG) analysis was performed using a Pyris1 instrument analyser (Perkin-Elmer, Sheffield, UK) to monitor the weight loss/gain and heat treatment processes under a flow of dry air with a 10 °C/min heating rate. The specific surface area was analysed by the Brunauer–Emmett–Teller (BET) method using Micromeritics ASAP 2010 in which the N_2_ gas adsorption was employed. The morphology and composition of the samples were investigated by scanning electron microscopy ZEISS model ULTRA 55 (Jena, Germany), equipped with an energy-dispersive X-ray spectrometer (EDX). HRTEM images were obtained using an electronic microscope JEOL model JEM-2010 (Pleasanton, CA, USA). The magnetic measurements (susceptibility and magnetization) were performed with two fully automated SQUID magnetometers (Quantum Design MPMS-5S, San Diego, CA, USA) in the temperature range 4–300 K, as described elsewhere [24].

### 2.3. Electrochemical Tests

Electrochemical tests were conducted on CR2025-type coin cells. The positive electrodes were constituted of 80 wt % active material, 10 wt % carbon black as conductive material and 10 wt % polyvinylidene fluoride (PVDF) in N-methyl pyrrolidinone (NMP) solvent, mixed and ground to form a homogeneous slurry. The slurry was then spread onto an aluminium foil current collector and dried at 80 °C for 2 h to remove the solvent before being pressed. The cathode loading was in the range 5–7 mg·cm^−2^. The cells were assembled in a glove box (moisture and oxygen content ≤5 ppm) under argon atmosphere using lithium sheet as the counter electrode, Celgard 2300 film (MTI, Richmond, CA, USA) as the separator, and 1 mol·L^−1^ LiPF_6_ in ethylene carbonate (EC)/diethyl carbonate (DEC) (1:1) solution as electrolyte (LP30, Merck, Darmstadt, Germany). The galvanostatic charge–discharge curves were performed using a potentiostat/galvanostat (VMP3 Bio-Logic, Claix, France) in the potential range 2.0–4.8 V.

## 3. Results and Discussion

### 3.1. The Effect of Particle Size

**Figure 4:**
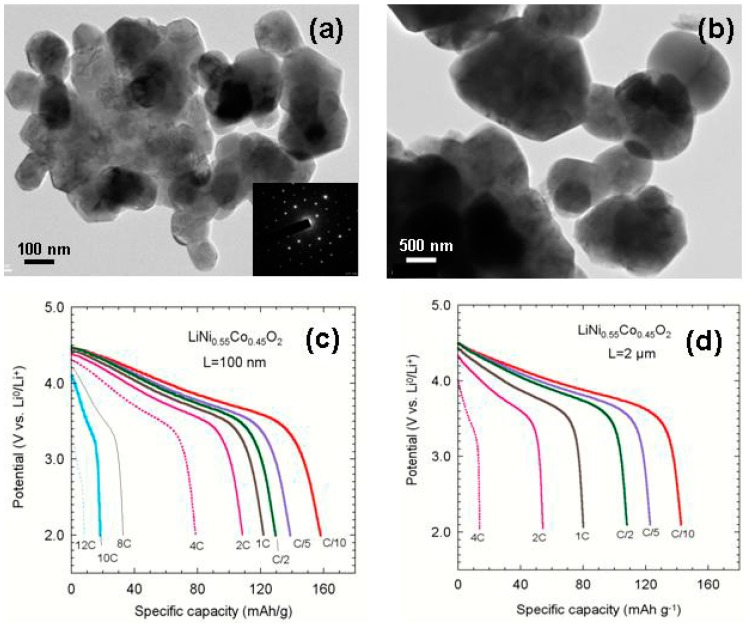
HRTEM images of LiNi_0.55_Co_0.45_O_2_ layered powders with different particle sizes: ca. 100–150 nm (**a**) and 1.5–2.0 µm (**b**) and discharge profiles as a function of C-rate for Li//LiNi_0.55_Co_0.45_O_2_ coin-type cells with two cathode materials of 100 nm and 2 µm particle size. The good crystallinity of powders is shown by the electron diffraction diagram (inset); (**c**) discharge curves for 100–150 nm; (**d**) discharge curves for 1.5–2.0 µm.

The main drawback of layered of TMOs is their poor discharge rate capability due to low intrinsic electronic and ionic conductivity. Thus, at high current densities, i.e., *J* > 1C rate (the rate is denoted C/n, where C is the theoretical cathode capacity and a full discharge occurs in n hours), the poor electrochemical performance is attributed to the low electron transport of the material and the slow Li-ion kinetics within the grains. The currently adopted approach to get high rate capability is to reduce the diffusion path length of charge species by minimizing the particle size of the active phase [25]. For instance, Okubo et al. [26] have observed an excellent high-rate capability, i.e., 65% of the 1C rate capability at 100C, in nanocrystalline LiCoO_2_ with an appropriate particle size of 17 nm. In this context, based on the Li-ion diffusion coefficient D* ≈ 2.5 × 10^−12^ cm^2^·s^−1^, the discharge process of 100 s requires particle size *L* = 100 nm against 1 h for *L* = 2 µm. Thus, 100-nm sized particles can be fully charged/discharged even at 10C rate (1.4 A·g^−1^). It was also demonstrated that control of the particle size is obtained via synthetic methods such as sol-gel [27], hydrothermal process [28], etc. As an example of the particle size effect, LiNi_0.55_Co_0.45_O_2_ (NCO) compounds were investigated. Figure 4 shows the HRTEM images of NCO particles and the discharge capacity curves of the corresponding Li//LiNi_0.55_Co_0.45_O_2_ coin-type cells as a function of C-rate. The cathode material (a) prepared by hydrothermal method has nanometric particles, 100–150 nm average size, while the micron-sized material, 1.5–2.0 µm particle size, was prepared by a two-step co-precipitation technique. An obvious difference in the electrochemical performance is observed. The Li cell with nano-sized particles allows a specific capacity 150 mAh·g^−1^ for 1C discharge rate, which is twice the capacity of the cell with micro-sized particles. The excellent rate capability makes nano-LiNi_0.55_Co_0.45_O_2_ suitable for high-power LIBs. Note that battery cycle life is favored by the architecture of the cathode material at the sub-micron scale, which allows the accommodation of volume changes caused by Li^+^ ions insertion/extraction into/from the single particle due to faster stress relaxation.

### 3.2. The Effect of Cationic Mixing

**Figure 5:**
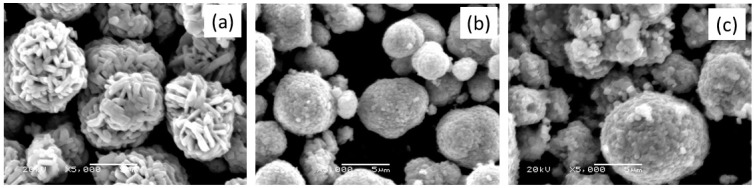
SEM images of NMC samples: (**a**) 333; (**b**) 442; and (**c**) 532 compounds prepared by solid-state reaction at 850 °C in air.

In this section, we report the properties of mixed transition-metal oxides LiNi_w_Mn_y_Co_z_O_2_ as 4-volt cathode materials for Li-ion batteries, for which the electrochemical features are correlated with structure and morphology of active particles. Special attention is given to the influence of the cation mixing between Li^+^ and Ni^2+^ ions on the crystallographic (3*b*) sites of the layered lattice due to the fact that the ionic radius of Ni^2+^ (0.69 Å) close to that of Li^+^ (0.76 Å) in an octahedral environment. A partial occupation of Li(3*b*) sites generates a disorder, so-called “cation mixing”, in the structure that damages the electrochemical performance [29,30,31]. Figure 5 presents the SEM images of three NMC samples prepared by solid-state reaction at 850 °C in air with composition 333, 442, 532. The surface morphology of NMC particles displays powders formed by aggregates of primary particles, which appear as small column-shaped particles. Typical dimensions of the small columned particle are 0.5 µm × 2 µm for the 333 compound. The SEM images of samples 442 and 532 display spherical secondary powders formed by aggregates of primary particles, which appear as small cubic-shaped particles. The formation of spherical secondary particle is beneficial for achieving a high tap density and energy density. In addition, it should be noted that, in cathode materials, the Li^+^ ion diffusion not only depends on the size of primary particles but on the morphology and porosity of the secondary particles.

**Figure 6:**
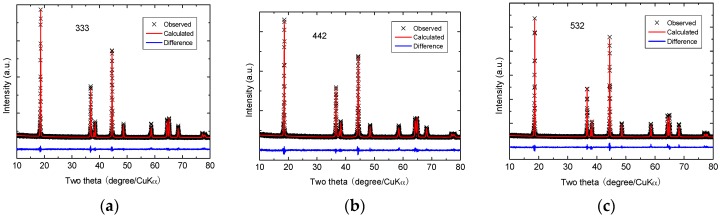
Rietveld refinement patterns of NMC samples: (**a**) 333; (**b**) 442; (**c**) 532 synthesized by solid-state reaction. The cross marks show observed XRD intensities and the solid line (red) represents calculated intensities. The curve at the bottom (blue) is the difference between the calculated and observed intensities on the same scale.

Structural investigations (X-ray diffraction and Raman spectroscopy) show that the samples calcined at 850 °C exhibit the typical rhombohedral *α*-NaFeO_2_ structure ($R\bar{3}m$ S.G) without any other impurity phases. XRD data were refined by the Rietveld method, using the structural model (Li_1-*δ*_Ni*_δ_*)_3*b*_(Li*_δ_*Ni_x-*δ*_Mn_y_Co_1-x-y_)_3*a*_O_2_ (see details in [32]). The results are shown in Figure 6 and the data refinements are listed in Table 1. The cation mixing corresponds to an antisite defect noted Ni_Li_, which means that Ni^2+^ occupies a Li^+^ site that is compensated for by the opposite, i.e., Li’_Ni_, corresponding to a Li^+^ ion on a Ni^2+^ site preferentially close to it, to result in a neutral Ni_Li_ + Li’_Ni_ pair, with one Ni^2+^ ion on a 3*b* site plus one Li^+^ on a 3*a* site. The fraction of Li^+^ on 3*a* sites, which is the concentration of such antisite defects, is called *η.* Indeed, as expected, the concentration of Ni(3*b*) defects and then the Li^+^/Ni^2+^ cation mixing is less (*η* = 1.56%) for the 333 compound. The *c*/*a* value is related to the degree of trigonal distortion, a higher cation ordering being achieved when *c*/*a* > 4.899. The evolution of this ratio with *z* in Table 1 indicates that the substitution of Co for Mn in LiNi_w_Mn_y_Co_z_O_2_ promotes the formation of the layered lattice [30]. The trigonal distortion *c*/*a* is largest for the 333 compound and lowest for the 532 one, which indicates that a smaller concentration of Ni results in a better layered ordering and smaller concentration of Li^+^/Ni^2+^ cation mixing.

The cation mixing would attract oxygen ions from adjacent oxygen layers, thereby locally shortening the oxygen–oxygen distance between neighbouring *M*O_2_ (*M* = TM ions on 3*b* layer) slabs. Moreover, the Li-ions migrating between two neighbouring octahedral sites in the same lithium layer are also required to pass through an empty tetrahedral site. The decrease in the oxygen–oxygen distance would increase the activation energy for the Li-ion motion [32]. Furthermore, the Ni^2+^(3*b*) ions would exert a strong electrostatic repulsion on the Li ions and drastically hamper their reinsertion. The sample with a higher concentration of cation mixing would induce very high Li diffusion barrier in less-ordered materials, resulting in its relatively worse electrochemical performance. For 442 and 333 compounds, the charge compensation is achieved by stabilization of Ni^2+^, Mn^4+^, and Co^3+^. Due to the excess of nickel-ion in the 532 compound, the charge neutrality requires the oxidation of Ni^2+^ (0.2 mol per chemical formula) to Ni^3+^. The amount of cation mixing estimated from magnetic measurements is consistent with the XRD results.

**Figure 7:**
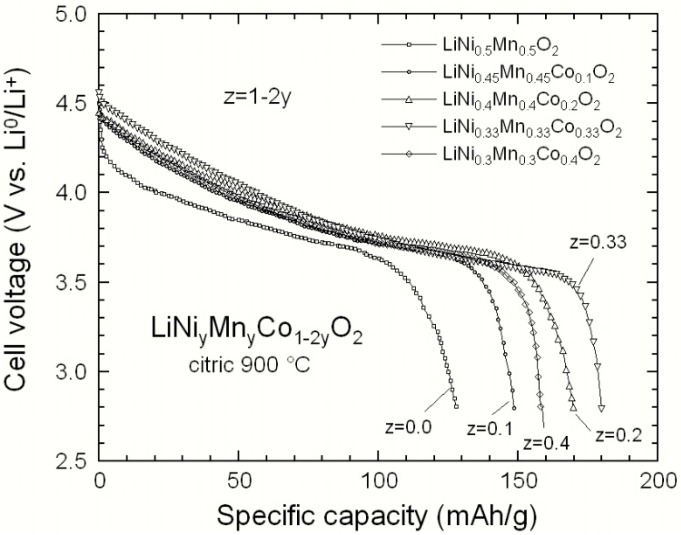
Typical discharge profiles of different Li//NMC cells with cathodes synthesized by the solid-state reaction route at 850 °C. The electrochemical test were carried out at discharge rate C/5 in the voltage window 2.8–4.6 V vs. Li^0^/Li^+^.

Figure 7 displays the typical discharge profiles of different Li//NMC cells with cathodes synthesized by the solid-state reaction method. The electrochemical tests were carried out at discharge rate C/5 in the voltage window 2.8–4.6 V vs. Li^0^/Li^+^. The dependence of the specific capacity on the composition is clearly seen. We observe smooth discharge curves with an average voltage near 3.80 V. The best result is obtained for the symmetric 333 compound, which exhibits a small irreversible capacity loss (5.5 mAh·g^−1^) at the first cycle, against 22 mAh·g^−1^ for the LiNi_0.5_Mn_0.5_O_2_ electrode. The discharge curves show the typical one-step behaviour characteristic of these layered compounds. These results illustrate the drastic change due to the introduction of a fraction of Co in the LiNi_y_Mn_y_Co_1-2y_O_2_ lattice. The 333 cell discharged at C/5 delivers a reversible capacity of ca. 180 mAh·g^−1^ with a capacity retention of 88% after 50 cycles, while 72% is obtained for the 550 compound. The negative effects of cation mixing *η* and decrease of the oxygen–oxygen distance on the electrochemical properties, which we have discussed above, is well evidenced by comparing the results in Figure 7 and in Table 1. For instance, the capacity delivered by the 442 sample is smaller than that of the 333 sample, because the concentration of antisite defects *η* is larger.

**Figure 8:**
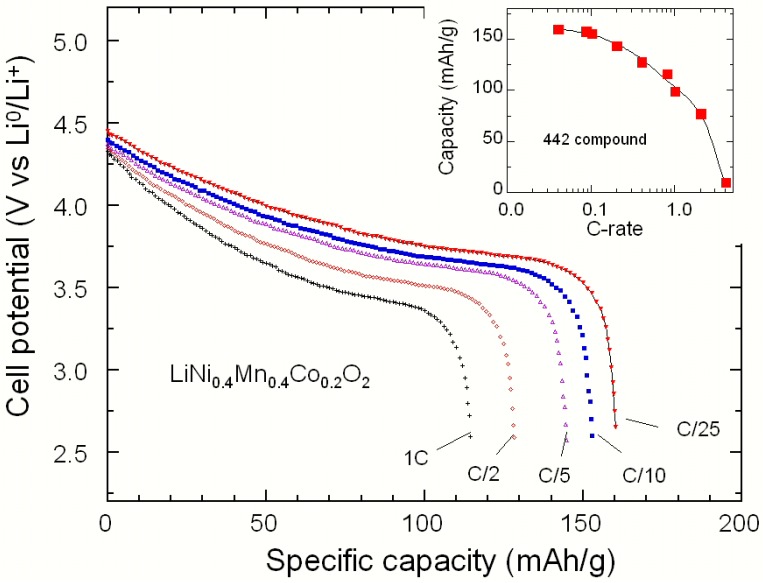
Discharge profiles vs. specific capacity of the Li//LiNi_0.4_Mn_0.4_Co_0.2_O_2_ cells between 2.5 and 4.4 V at various C-rates. The inset shows the rate capability of the 442 compound.

As a typical example of an electrochemical test, Figure 8 presents the discharge profiles as a function of C-rate for the Li//LiNi_0.4_Mn_0.4_Co_0.2_O_2_ coin cell cycled in the potential range 2.5–4.4 V vs. Li^0^/Li^+^. This is a typical illustration of the rate capability of the 442 compound grown by wet chemistry, which is considered as having the highest capacity and maintaining its capacity on cycling [33,34,35,36]. The cell discharged at C/25 delivers a reversible capacity of 160 mAh·g^−1^, with a coulombic efficiency of 88% at the first cycle. These data are similar to those reported by Tran et al. for LiNi_0.425_Mn_0.425_Co_0.15_O_2_ [34]. Upon further cycling, the capacity retention remains quite stable despite a rather large amount (2.71%) of Ni^2+^ antisites in the lithium interslab space of the 442 material. The continuous fall of the capacity against the increasing current density is attributed to the low electronic conductivity of 1.4 × 10^−4^ S·cm^−1^ for LiNi_0.4_Mn_0.4_Co_0.2_O_2_ [35]. The good electrochemical performance has also been pointed out by Tsai et al. [37] from X-ray absorption spectroscopy. The inhibition of structure distortion during the delithiation and lithiation cycle originates from a balance of the repulsive force and the size effect in the layered compound. Upon further cycling, the capacity retention remains quite stable despite a rather large amount (ca. 3%) of Ni^2+^ in the interslab site of the 442 material. Cycling with the upper potential of 4.5 V, the cell delivered a capacity of about 170 mAh·g^−1^ at a specific current of 40 mA·g^−1^ (C/15). The tests of electrochemical performance and limit of discharge rate in NMC were obtained from the Peukert plot (insert in Figure 8), which revealed that the 442 compound delivers almost half of the initial capacity at 2C rate.

The open-circuit curve or the discharge profile at low rate (solid line at C/25 in Figure 8) for the Li//LiNi_0.4_Mn_0.4_Co_0.2_O_2_ cell can be modelled by the equation:

(4) $$E\left(x\right)=\sum_{n=1}^{i}\left(E_{i}^{o}+\frac{RT}{F}\ln\frac{x_{i}}{x_{\max}-x_{i}}+\frac{RT}{F}K_{i}x_{i}\right)$$

The fitting parameter *K_i_* reveals the interactions between the inserted Li^+^ and the available sites of the host. The parameter *K* is a lateral interaction parameter that is introduced to represent the effects of the interactions in a two-dimensional process: *K* > 0 for a repulsive interaction, *K* = 0 for an absence of any interaction, 0 > *K* > −4 for attractive interaction between the intercalation sites, *K* = −4 is a critical state, while for *K* < −4 the interactions are so intensive that they lead to the existence of two-phase reactions. 

For NMC insertion compounds, we consider two redox processes labelled by the index I = 1,2. The first process is associated to the valence change of the nickel ions: Ni^2+^ → Ni^3+^ → Ni^4+^. The first step of delithiation extends from the lithium concentration of lithium on 3*b* sites:

*x*_max,1_ = 1 (5)

until the Ni ions have been converted into Ni^3+^, in which case *x* = 1 − *y*. In Equation (5), we have neglected the amount of lithium on the 3*a* sites, which is justified by the fact that the concentration of antisites is few per cent only. Then, Ni^3+^ ions can be converted to Ni^4+^ until *x* = 1 − 2*y*, where all the nickel ions have been converted in the Ni^4+^ valence state. At this concentration a second redox process Co^3+^ → Co^4+^ begins, which extends from

*x*_max,2_ = 1 − 2*y* (6)

down to *x* = 0, where all the cobalt ions have been converted to the tetravalent state. Note we have implicitly assumed that the different processes do not overlap. This has been justified in [38], where we have shown from the analysis of magnetic measurements that the concentration *y*′ of Ni^3+^ at the concentration *x*_max,2_ does not exceed 2%, which is the uncertainty in the determination of *y*′.

The best fit (Figure 8) for the 442 cathode material is obtained with two redox processes with standard potential *E*_0,1_ = 3.731 V and *E*_0,2_ = 3.955 V and interaction factor *K*_1_ = −3.75 and *K*_2_ = −2.98. Since the interaction is smaller than the critical case *K*_c_ = −4 we are not in the case of a two-phase regime, and indeed there is no plateau in the curves in Figure 7 and Figure 8. Table 2 lists the electrochemical parameters of NMC materials studied in Figure 8.

However, we know from Table 1 that the cation mixing decreases linearly with the concentration of cobalt 1 − 2*y* from *η* = 7% for (1 − 2y) = 0 to 0.7% for (1 − 2*y*) = 0.5. While the increase of cation mixing has important effects on the electrochemical properties that we have evidenced in Figure 8 for instance, we find here that it has very little effect on the parameters in Table 2. The reason is that the increase of the cobalt concentration increases the electrical conductivity and the correlated decrease of the cation mixing increases the ionic conductivity, hence the beneficial impact on the electrochemical properties. On the other hand, the parameters in Table 2 are parameters of the redox reactions that are only sensitive to the average crystallographic environment of the nickel and cobalt ions, which is not significantly disturbed by few % cation mixing in the lattice.

### 3.3. Adjustment of the Li/M Ratio

**Figure 9:**
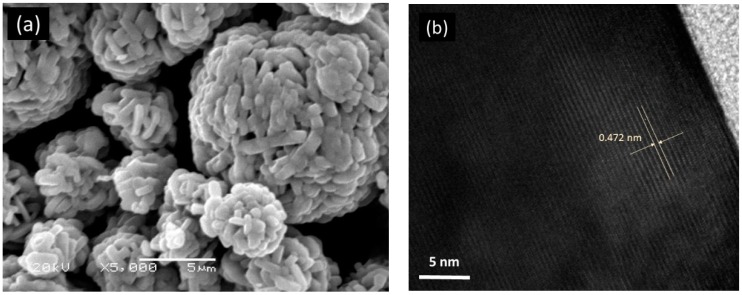
SEM (**a**) and HRTEM (**b**) images of Li_1.04_Ni_0.32_Mn_0.32_Co_0.32_O_2_ powders synthesized the co-precipitation method from hydroxide precursor assisted by ammonium and sodium hydroxides.

Another way to optimize an electrode active material consists in the adjustment of one parameter of the synthesis, namely the lithium/transition metal molar ratio in Li_1+x_(NMC)_1-x_O_2_ (NMC = 333) to minimize the cation mixing [30]. Two independent experimental procedures are used to estimate the cation mixing (*η*): structural refinements of XRD patterns made by the Rietveld method, which accurately determine the *M* occupancy between slabs of the layered lattice; (ii) magnetic measurements, which are powerful to check the quality of material and structural properties at the nano-scale [39] of samples synthesized by the co-precipitation method from a hydroxide precursor assisted by ammonium and sodium hydroxides [40]. The elemental titration carried out by ICP of the two samples prepared by a hydroxide route with nominal Ψ = Li/*M* ratio (Ψ = 1.05 and 1.10) shows the technological composition of Li-rich Li_1+x_(Ni_1/3_Mn_1/3_Co_1/3_)_1-x_O_2_ with x = 0.02 and 0.04, respectively. Note that the Li/*M* ratios of the samples are (1 + x)/(1 − x) = 1.04 and 1.08, respectively, close to the nominal ratio Ψ. This gives evidence that the amount of Li that has evaporated during the synthesis is very small (1%–2% only), and comparable to the case when the samples are prepared by the Pechini method [40]. The morphology of particles is shown in Figure 9. The SEM micrograph displays powders formed by agglomerates of primary particles, which appear as small, column-shaped particles of typical dimension 0.5 µm × 2 µm. The HRTEM image shows the good crystallinity of the individual nanoparticles. Apparent lattice fringes can be observed and the width of 0.472 nm between lattice fringes is consistent with the interspacing of the (003) planes of the layered structure $R\bar{3}m$ space group.

If *u* is the concentration of Ni*_Li_ + Li’_Ni_ pair defects, the detailed chemical formula is then (Li_1-u_Ni_u_)_3b_(Li_x+u_Ni_(1-x)/3-u_Mn_(1-x)/3_Co_(1-x)/3_)_3a_O_2_ for Rietveld analysis: both *x* and *u* were refined, from which the fraction *η* of Li on 3*a* sites has been deduced. The crystallographic parameters are listed in Table 3. In particular, *y* is found to be lower in sample Li_1.04_Ni_0.32_Mn_0.33_Co_0.33_O_2_, a result in good agreement with the value obtained from analysis of magnetic measurements.

**Figure 10:**
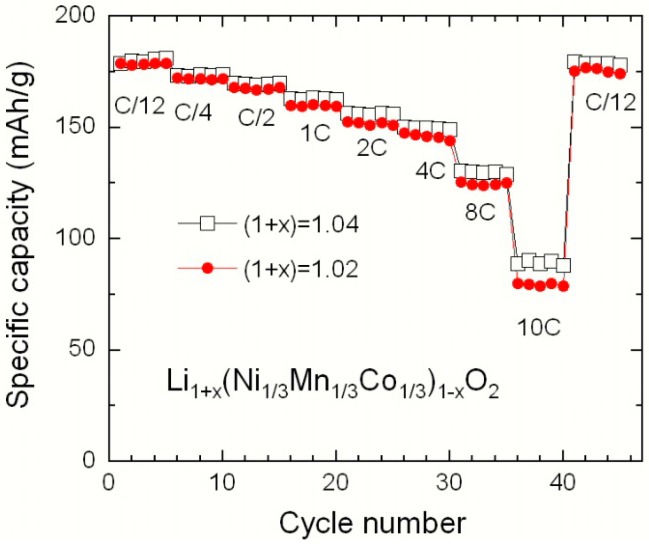
Electrochemical performance of Li_1+x_(NMC)_1-x_O_2_ electrode materials as a function of synthetic parameters. The specific capacity of the Li cells is plotted for different C-rates.

Figure 10 displays the discharge capacity of the Li_1+x_(NMC)_1-x_O_2_ cathode materials as a function of the cycle number with increasing discharge rate from C/12 to 10C. The electrode material prepared with a Li excess of 4% exhibits the best performance at high C-rate. These electrochemical features show that the cation mixing has a slight effect on the discharge behaviour attributed to kinetic hindrance from Ni^2+^(3*b*) ions in the Li layer. Note the superior discharge capacity of the 333 compound compared to that of 442 one, since the increase in the Co content in NMC favours the Li^+^ motion in the crystal lattice by improving the electronic conductivity of the electrode. In conclusion, a larger excess of lithium in the product counteracts the cation mixing (smaller value of *y*) and thus reduces the concentration of ferromagnetic Mn^4+^(3*a*)–Ni^2+^(3*b*) interactions. This is actually a general trend in layered compounds, since it has been observed also in Li_1+x_(Ni_0.5_Mn_0.5_)_1-x_O_2_ [41].

### 3.4. Effect of the Chelating Agent

With a large amount of un-removable Ni ions in the Li(3*b*) site that block the Li diffusion pathways, the Li^+^-ion mobility in the host lattice is negatively affected. For example, it was reported that the chemical diffusion coefficient of Li in LiNi_0.5_Mn_0.5_O_2_ (i.e., 3 × 10^−10^ cm^2^·s^−1^) is lower than in LiCoO_2_ (i.e., 5 × 10^−9^ cm^2^·s^−1^) resulting in the intrinsically low rate capability of this electrode material [42,43]. In this section, we investigated the effect of the transition-metal/chelating agent molar ratio, *R* (with 0.5 ≤ *R* ≤ 5) on the structural and electrochemical properties of the symmetric 333 NMC materials synthesized by the co-precipitation method assisted by succinic acid (dicarboxyl C_4_H_6_O_4_).

**Figure 11:**
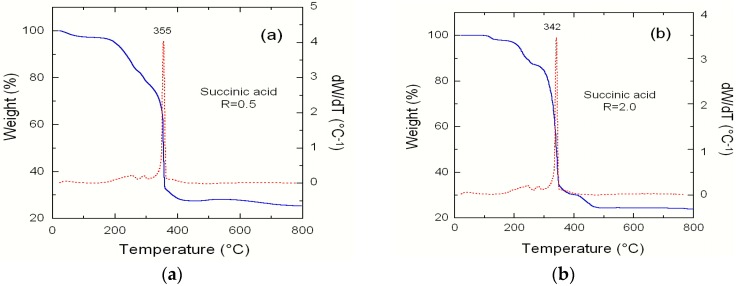
(**a**) TG/DTA profile for the precursor of NMC synthesized by the co-precipitation technique using metal acetates as raw materials and succinic acid as a polymeric agent with a chelate to metal ion molar ratio *R* = 0.5 (**a**) and *R* = 2 (**b**). With *R* = 2, the combustion temperature is lowered to 342 °C.

The TG/DTA profiles for the mixed precursor of NMC material synthesized via *R* = 0.5 are presented in Figure 11. Several weight loss stages are observed in this TG curve. The first mass loss stage occurs at *T* < 300 °C, which can be assigned to the loss of absorbed water, the residual ethanol, the dehydration of metal acetate, and the crystal water. The anhydrous metal acetate can be decomposed into both metal oxide and gases such as carbon mono- and di-oxide by further thermal treatment in air [13]. The weight loss in the second step is observed around 355 °C, which corresponds to the decomposition of succinic acid and acetate ions xerogel. For *R* = 2, the combustion temperature has decreased to 342 °C, which produces smaller crystallites. A weight loss of 72.4% occurs during these two stages because of the oxidation–decomposition reaction. It appears that tartaric acid acts as a fuel in the pyrolysis of the gel precursor, favouring the decomposition of acetate ions. After 450 °C, there is little weight loss, so in this work we choose 450 °C as the heating temperature for the pre-calcination.

**Figure 12:**
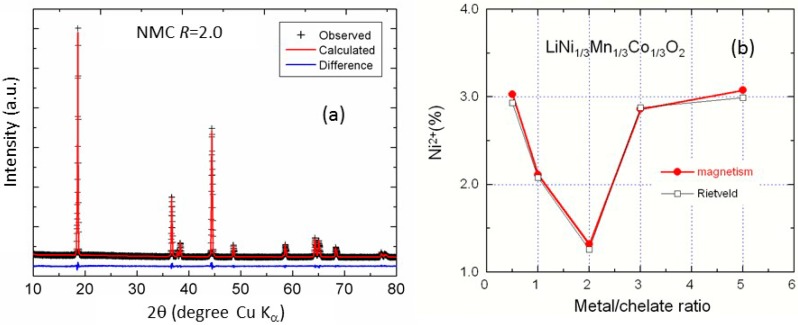
(**a**) Rietveld refinement patterns of NMC synthesized at *R* = 2 via wet chemical method (*R*_p_ = 9.8%). The plus marks show observed X-ray diffraction intensities and the solid line (in red on the web version) represents calculated intensities. The curve at the bottom (in blue on the web version) is the difference between the calculated and observed intensities on the same scale; (**b**) The concentration of Ni(3*b*) defects (site-exchange rate of the Li^+^/Ni^2+^ cation mixing) as a function of the *R* parameter for the symmetric NMC compounds.

Figure 12a shows the typical Rietveld fit of the XRD pattern for the sample *R* = 2. The refinement was performed using the model (Li_1-*δ*_Ni*_δ_*)_3*b*_(Li*_δ_*Ni_x-*δ*_Mn_y_Co_1-x-y_)_3*a*_O_2_ [34]. Indeed, the concentration of Ni(3*b*) defects and then the site-exchange rate of the Li^+^/Ni^2+^ cation mixing is as low as 1.26% for the *R* = 2 compound, as presented in Figure 12b. With the highest *c*/*a* ratio and the smallest unit volume cell, the *R* = 2 sample exhibits the best layered structure. These results follow the variation of the crystallographic parameters (Table 4). The minimum of site-exchange rate corresponds to a minimum of the unit-cell volume and a maximum of degree of trigonal distortion given by the *c*/*a* ratio that indicates a higher cation ordering being achieved.

**Figure 13:**
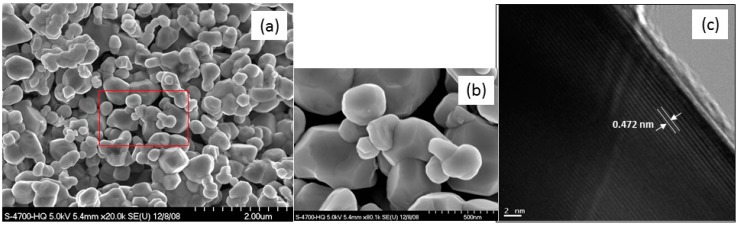
(**a**,**b**) SEM images of NMC synthesized at *R* = 2 via wet chemical method at different magnifications; (**c**) HRTEM image showing the fringe system corresponding to the spacing between (003) lattice planes in the $R\bar{3}m$ space group.

The study of the morphology clearly proved that the NMC synthesized with *R* = 2 presents the best crystallinity, consistent with the XRD results. The chelating agent (in our case carboxylic acid) determines the acidity of the solution. The R value must be large enough to obtain a weak acid solution necessary to the crystallization of the oxide material. However, it must be small enough to avoid direct action with the raw materials (acetates or nitrates). That is in essence the reason why there is an optimum value for this parameter. Figure 13 displays the SEM and HRTEM images of this sample. The patterns provide evidence for the structural quality of the samples examined. Image (a) shows the representative SEM pattern of NMC powders that indicates a material containing spherical shape crystallites, as confirmed by the bright-field low-magnification TEM. Primary particles of ~0.3–0.5 µm size are regularly distributed. However, at a smaller scale (image b), relatively clear facets can be observed at the surface of some primary particles. The HRTEM pattern of an NMC particle (image c) shows a set of acute parallel fringes extending to the particle edge. Careful observation indicates that the fringes show a spacing distance of 0.472 nm, which corresponds to the spacing between (003) lattice planes that diffract the X-rays at angle 2*θ* = 18.7°. It is worth noting that the good morphology of the *R* = 2 sample is considered to be favourable for obtaining high tap density and enhancing the electrochemical performance, i.e., improving the kinetics of lithium ions.

**Figure 14:**
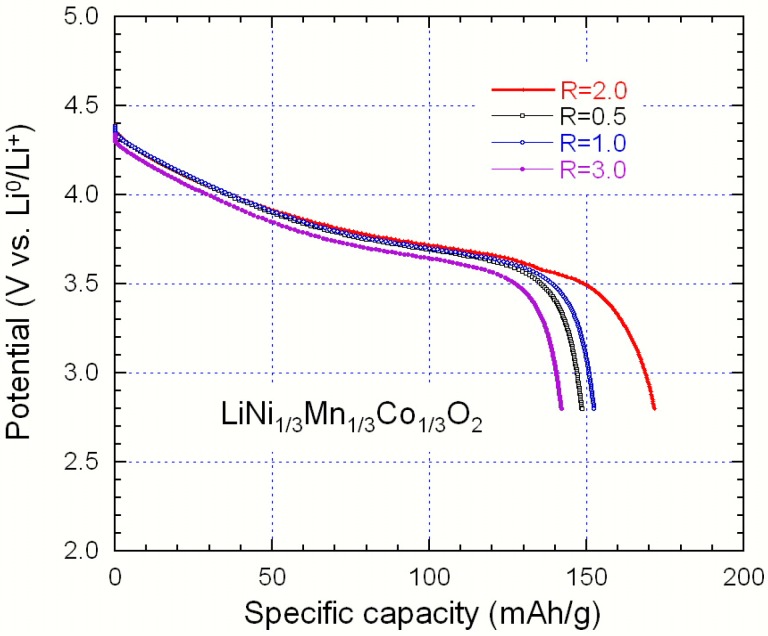
Initial discharge profiles of synthesized NMC sample sintered at 900 °C for 15 h, synthesized with chelate to metal ion ratio at 0.5 ≤ *R* ≤ 3.0.

Figure 14 presents the initial discharge profiles of NMC samples sintered at 900 °C for 15 h, synthesized with an acid to metal ion ratio at 0.5 ≤ *R* ≤ 3.0. The charge and discharge processes were carried out at a constant current density of 16 mAh·g^−1^ (0.1 C rate) in the potential range of 2.8–4.4 V vs. Li^0^/Li^+^ at room temperature. As expected from the structural investigations, the NMC *R* = 2 material displays the best performance. Its lower cationic disorder results in its higher discharge capacity. The first charge and discharge capacity are 184 and 172 mAh·g^−1^, respectively. The irreversible capacity loss in the first cycle is 12 mAh·g^−1^ and the coulombic efficiency is 93.5%. After four cycles, the discharge capacity is 168 mAh·g^−1^ and 97.7% of the first discharge capacity has been retained. Therefore, the rather small coherence length of the lattice does not affect the good electrochemical performance. The electrochemical storage energy and intercalation mechanism in NMC in the potential range limited to 2.80–4.45 V are attributable to the Ni^2+^/Ni^4+^ redox reaction pair.

### 3.5. Effect of the Synthesis Recipe

**Figure 15:**
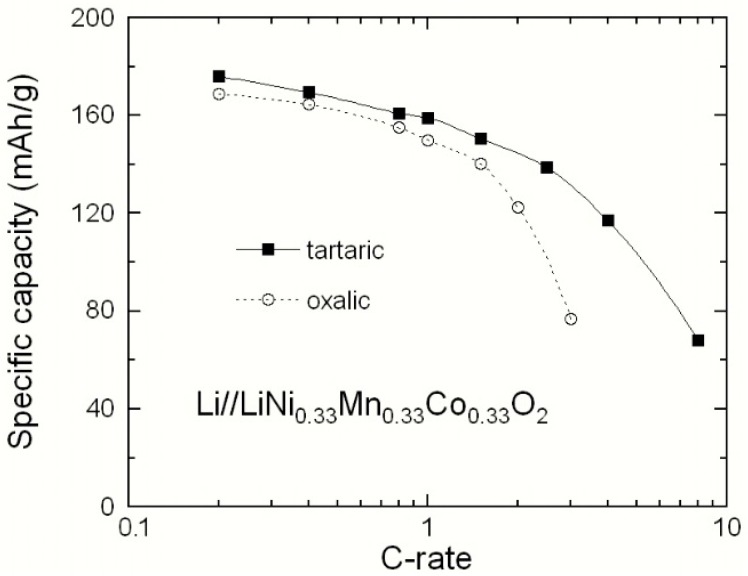
Peukert plots showing the difference in the electrochemical performance of LiNi_1/3_Mn_1/3_Co_1/3_O_2_ electrodes synthesized using wet-chemical method assisted by oxalic and tartaric acid.

The synthesis parameters such as the nature of the chelating agent and the annealing temperature can strongly influence the growth process of NMC particles and thus their structure and morphology. As an example, Figure 15 presents the Peukert plots that show the difference in the electrochemical performance of LiNi_1/3_Mn_1/3_Co_1/3_O_2_ electrodes synthesized using a wet-chemical method assisted by oxalic (single dicarboxyl) and tartaric (complexed dicarboxyl) acid. It was reported that the chelating agent (carboxylic-based acid) provoked decomposition during the synthesis of oxide powders and the gel precursor was burning because the decomposed acetate ions acted as an oxidizer [44]. The wet-chemical synthesis techniques are based on the sample preparation via inorganic polymerization reactions in solution. The methods consist of an acidification reaction using organic (carboxylic) acids as complexing agents (chelate) like citric, succinic, oxalic, and tartaric acids including the –COOH– carboxyl groups that act as fuel during the formation process of electrode powders.

**Figure 16:**
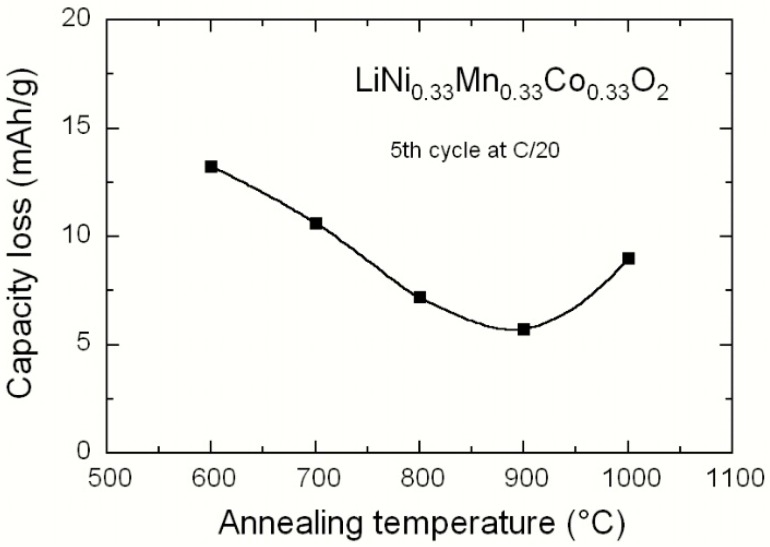
Initial capacity loss of LiNi_1/3_Mn_1/3_Co_1/3_O_2_ electrodes cycled at C/20 as a function of the annealing temperature.

Processing at high temperature is usually necessary to oxidize the TM ions under an oxygen atmosphere to obtain a high degree of crystallinity. However, the annealing process needs to be carefully conducted because high temperature favours (i) the growth of large crystallites, which is not desirable for use in high power density applications requiring nano-sized particles and (ii) a loss of lithium oxide, Li_2_O, due to its volatility that induced sub-stoichiometric powders. Figure 16 shows the variation of the initial capacity loss of LiNi_1/3_Mn_1/3_Co_1/3_O_2_ material synthesized by the tartaric acid route as a function of the annealing temperature, *T*_a_. The electrodes were cycled at C/20 (~10 mA·g^−1^). The minimum of capacity loss is obtained for *T*_a_ ≈ 900 °C, which corresponds to an optimized particle with good crystallinity and morphology for electrode function, but also to an ordered lattice with minimum cation mixing.

### 3.6. Effect of Small Deviation of Co Content

**Figure 17:**
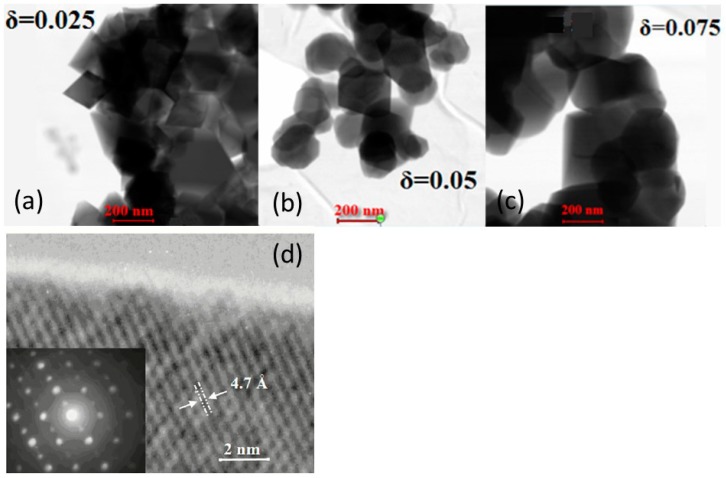
(**a**–**c**) TEM images of individual LiNi_0.33+*δ*_Mn_0.33+*δ*_Co_0.33-2*δ*_O_2_ nanoparticles with *δ* = 0.025, 0.05, and 0.075, respectively; (**d**) HRTEM image of a nanoparticle (*δ* = 0.025) showing lattice fringes consistent with the interspacing of the (003) planes of the $R\bar{3}m$ layered structure.

In this section, we investigate the deviation of the local environment of cations for different compositions LiNi_0.33+*δ*_Mn_0.33+*δ*_Co_0.33-2*δ*_O_2_ (0.025 ≤ *δ* ≤ 0.075) synthesized by sol-gel citrate route. Note that the use of citric acid (tricarboxyl) is beneficial to generate a gel instead of oxalic acid (dicarboxyl), which produces a precipitate. Single phased oxides with the typical layered rhombohedral structure were obtained after firing at 900 °C for 24 h. Figure 17 displays the TEM images (a–c) of NMC samples with an average particle size of about 200–350 nm, indicating that each particle is composed of a few crystallites (<*L*> = 70 nm). The change of particle morphology is clearly evidenced by the TEM pictures; we observed octahedron-like (*δ* = 0.025) and spherical like particles (*δ* = 0.05 and 0.075). An HRTEM image (Figure 17d) of a NMC nanoparticle displays bright- and dark-band contrasts with widths of a few nm. In each band contrast, there is a fringe contrast with a spacing of 0.47 nm that corresponds to the inter-planar spacing of the (003) hexagonal plane. The electron diffraction pattern (inset) confirms the crystallization in the $R\bar{3}m$ rhombohedral structure. The regularity in the lattice fringes implies that no dislocation is detected in the layered structure. As envisaged from our HRTEM studies, the localized domain-like features are only observed in nano-scale. The specific surface area *S_BET_* slightly changed with the increasing cobalt content from 2.42 to 2.76 m^2^·g^−1^, which confirms the particles’ size evolution evidenced by TEM analysis.

**Figure 18:**
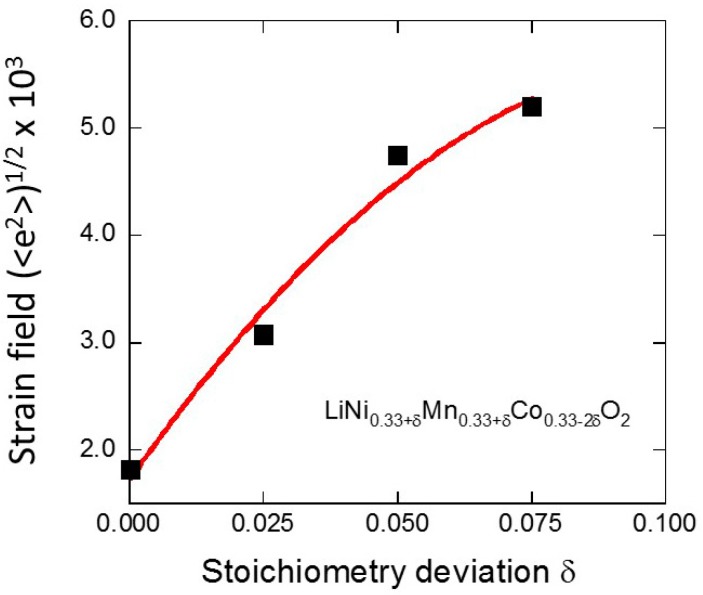
Evolution of the strain (<*e*^2^>)^1/2^ with the deviation from stoichiometry *δ* in LiNi_0.33+*δ*_Mn_0.33+*δ*_Co_0.33-2*δ*_O_2_ samples synthesized by the sol-gel method.

Rietveld refinements of XRD patterns show that the cation mixing is lower than 2.2% in all samples owing to the stabilization of the layered structure by the cobalt. XRD results suggest that the deformation of a lattice is associated with the substitution of (Ni, Mn) for Co, which is only local in nature. On another hand, this local deformation generates local strain fields that are responsible for a broadening of the XRD lines. Indeed, as the cobalt concentration decreases, the width of the XRD lines increases, so that the splitting of the (006)/(102) and (108)/(110) Bragg lines could be observed for all samples. This is not surprising since cobalt tends to stabilize the hexagonal layered structure. The deviation from stoichiometry, *δ*, generates a strain, adding to the microscopic strain in the unit cell due to replacement of transition-metal ions in the intralayer block and to the cationic disorder, i.e., defects created by Ni^2+^ ions in the interlayer space. The profile of the Bragg lines is thus the combination of the Scherrer’s equation for crystallite size and the Bragg’s law for diffraction, according to the equation

(7) $$B^{2}\cos^{2}\theta =16\langle e^{2}\rangle \sin^{2}\theta +\frac{K^{2}\lambda^{2}}{L^{2}}$$

where *B* is the full-width at half-maximum (fwhm) in radian, *θ* is the diffraction angle, and *K* is a near-unity constant related to crystallite shape. The crystallite size *L* and micro-strain local <*e*^2^> are determined by using Equation (7). Indeed, the strain (<*e*^2^>)^1/2^ increases importantly with the stoichiometry deviation *δ*, as shown in Figure 18. On the other hand, we find that 60 ≤ *L* ≤ 80 nm for all the samples, so that the replacement of cobalt does not significantly affect the coherence length. This is further evidence that the LiCoO_2_-like framework is disturbed by the incorporation of a great quantity of Mn and Ni ions [45].

As mentioned above, the electrochemical performance of materials is subordinate to the electrode microstructure and morphology. Moreover, the high crystallinity of material improves its performance because insertion and de-insertion processes occur along specific crystallographic sites and directions.

**Figure 19:**
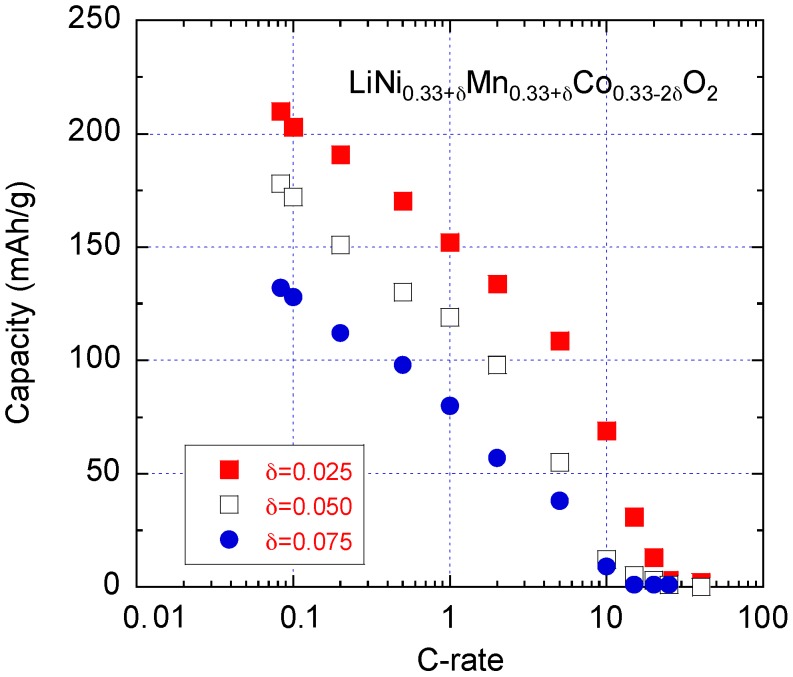
Peukert plots for Li//LiNi_0.33+*δ*_Mn_0.33+*δ*_Co_0.33-2*δ*_O_2_ cells.

The electrochemical tests showed that the positive electrode of composition *δ* = 0.025 displays a good electrochemical performance associated with the high stability of the layered framework. The discharge capacity decreases from 210 (C/12), 150 (1C) to 69 mAh·g^−1^ (10C) when cycled in the potential window 2.0–4.6 V vs. Li^0^/Li^+^, which shows that this material is well suited to develop high-powered batteries at the industrial scale for use in fast charge/discharge devices, e.g., HEV, due to its small concentration of cation mixing and high structural integrity [21]. Capacity retention of CE_1_: 82.3% and CE_2_: 95.2% was obtained with this electrode, which delivered a gravimetric capacity 197.8 mAh·g^−1^ at 90% DOD at C/5 rate. The rate performance of Li//NMC cells is depicted as Peukert plots in Figure 19. We observed a large decrease in the discharge capacity for high values of Co substitution (*δ* = 0.075), which is related with the increasing cationic mixing [46].

Note in Section 3.2 the samples were prepared with *δ* = 0. Among them the (442) sample delivered discharge capacity at C/5 is about 170 mAh·g^−1^. On the other hand, the samples in this section were prepared with *δ* ≠ 0. This is a significant difference between the 444 electrode material (Section 3.2) with the *δ* = 0.075 in the present section, which delivered only 110 mAh·g^−1^ at C/5. This result confirms that the deviation of *δ* from zero results in a degradation of the electrochemical properties, because it induces a strain field and a local deformation of the lattice quantified in Figure 18.

### 3.7. Layered-Layered Integrated Materials

In order to meet the demand for high energy density batteries, it is necessary to develop high-capacity cathode materials that are safe and present good electrochemical performance. For such a purpose, Li-rich oxides with the layered-layered integrated (LLL) configuration of composition *y*Li_2_MnO_3_•(1 − *y*)Li*M*O_2_, where *M* stands for Mn or a mixture of Mn with another transition metal that can be partially substituted for Mn, are of particular interest. The LLI materials have been the subject of extensive works that have been reviewed in [47]. However many problems still have to be solved. Current debates on structure and reaction mechanism, problems on electrochemical properties, and keys to the future study of these materials have been reviewed by Yu and Zhou [48].

In the following, we report experiments on Li(Ni_x_Li_(1/3-2x/3)_Mn_(2/3-x/3)_)O_2_, which can be rewritten in two-component notation as *y*Li_2_MnO_3_•(1 − *y*)LiNi_½_Mn_½_O_2_; it offers several advantages over conventional cathode materials: a specific capacity as high as 250 mAh·g^−1^, good structural stability, and high capacity retention at high voltage cutoff [49]. The choice of Ni doping is justified by the fact that Ni plays a positive role in the O^2−^ ions’ oxidation to O_2_ [50]. By extracting the Li ions with a concomitant oxygen release to form a layered MnO_2_ component at ca. 4.6–4.8 V vs. Li^0^/Li^+^, the Li_2_MnO_3_ component is considered to have two functions: (i) the stabilization of the electrode structure and (ii) the enhancement of the discharge capacity. In this section we report the study of *y*Li_2_MnO_3_•(1 − *y*)(LiNi_0.5_Mn_0.5_O_2_) samples with *y* = 0.0, 0.3 and 0.5 synthesized by citrate sol-gel method. These oxides are commonly viewed as integrated composites of layered Li_2_MnO_3_ with monoclinic *C*/2*m* symmetry and layered LiNi_0.5_Mn_0.5_O_2_ with rhombohedral $R\bar{3}m$ symmetry.

**Figure 20:**
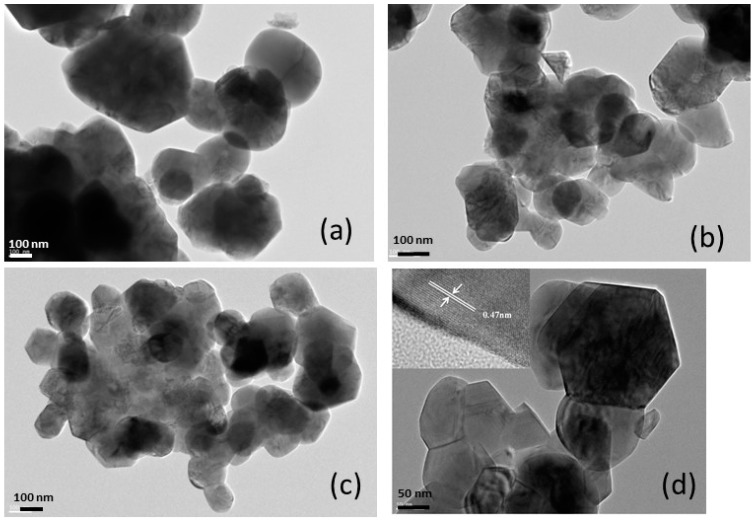
HRTEM images of Li-rich cathode materials *y*Li_2_MnO_3_•(1 − *y*)(LiNi_0.5_Mn_0.5_O_2_). (**a**) *y* = 0.0 (LiNi_0.5_Mn_0.5_O_2_); (**b**) *y* = 0.3 (Li_1.134_Ni_0.3_Mn_0.566_O_2_); (**c**) *y* = 0.5 (Li_1.2_Ni_0.2_Mn_0.6_O_2_); (**d**) image magnification for Li_1.2_Ni_0.2_Mn_0.6_O_2_ showing the hexagonal shape of nanoparticles.

Figure 20 shows the morphology of the LLL samples with *y* = 0.0, 0.3, and 0.5 synthesized by a citrate–gel method using acetate salts. These TEM images reveal highly ordered single crystalline particles with regular shape and morphology for all the samples. All the powders consist of primary particles smaller than 500 nm. The particles of the Li_1.134_Ni_0.3_Mn_0.566_O_2_ sample are smaller in size (~150 nm) and nearly monodisperse; however, a slight tendency to agglomerate is observed by SEM measurements (not shown here). The HRTEM micrograph (image d) shows that the Li_1.2_Ni_0.2_Mn_0.6_O_2_ powders are formed of well-crystallized hexagonal-shaped particles. The crystal faces are well defined and the distribution of particles is uniform.

In the inset (image d), one observes well-defined fringes corresponding to the typical *α*-NaFeO_2_ layered structure with interplanar distance of about 0.47 nm, which is the distance of the close-packed (003) planes of the rhombohedral $R\bar{3}m$ lattice (or the (001) planes of the monoclinic *C*2/*m* lattice). These fringes are observed over the whole region, which means that the pure single layered structured phase is built up throughout the careful synthesis recipe of wet chemistry. Analysis of XRD patterns show that the *c*_h_/*a*_h_ ratio increases linearly with the Li concentration. This effect is attributable to the increase of concentration of Mn^4+^ ions, the ionic radius of which (0.54 Å) is smaller than that of Ni^2+^ (0.69 Å) in octahedral coordination.

**Figure 21:**
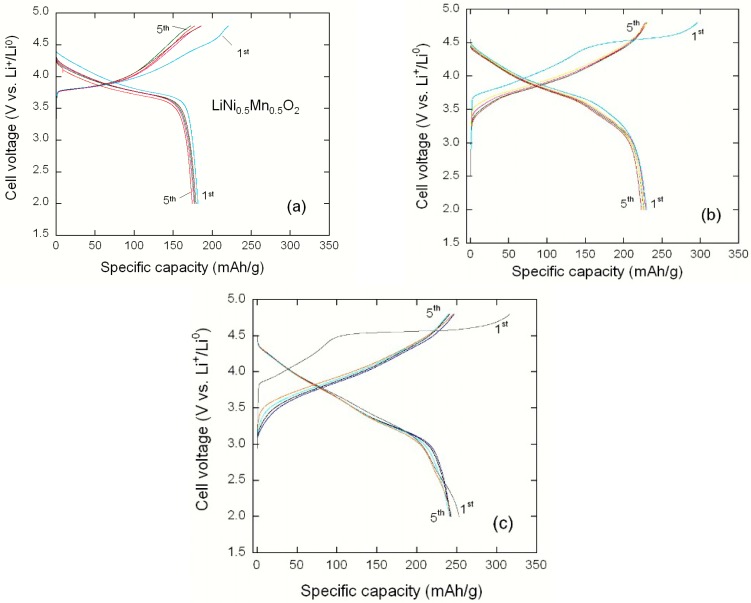
Charge–discharge profiles of Li//Li(Ni_x_Li_(1/3-2x/3)_Mn_(2/3-x/3)_)O_2_ coin cells: (**a**) LiNi_0.5_Mn_0.5_O_2_; (**b**) Li_1.134_Ni_0.3_Mn_0.556_O_2_; and (**c**) Li_1.2_Ni_0.2_Mn_0.6_O_2_. Cycles were carried out at C/10 rate (30 mA·g^−1^) in the potential range 2.0–4.8 V vs. Li^0^/Li^+^.

Figure 21 presents the electrochemical tests of Li//Li(Ni_x_Li_(1/3-2x/3)_Mn_(2/3-x/3)_)O_2_ coin cells cycled at C/10 rate (30 mA·g^−1^) in the potential range 2.0–4.8 V vs. Li^0^/Li^+^. The initial discharge capacity of the LiNi_0.5_Mn_0.5_O_2_ electrode is approximately 181 mAh·g^−1^ with a capacity loss of 39 mAh·g^−1^, while high discharge capacities of 230 and 253 mAh·g^−1^ are obtained for Li_1.134_Ni_0.3_Mn_0.556_O_2_ and Li_1.2_Ni_0.2_Mn_0.6_O_2_ samples, respectively. Upon increasing the lithium enrichment, the coulombic efficiency after the 2nd cycle slightly increases from 98.6% to 99.5%. On the contrary, the irreversible capacity loss in the first cycle slightly increases from 20 to 23 mAh·g^−1^ with the Li-rich oxides, i.e., Li_1.134_Ni_0.3_Mn_0.556_O_2_ and Li_1.2_Ni_0.2_Mn_0.6_O_2_, respectively. For all the cells, the first irreversible capacity associated with the high voltage plateau is due to the activation of the Li_2_MnO_3_ component with extraction of lithium and oxygen release at approximately 4.5 V. For subsequent cycles their coulombic efficiency is stabilized, in contrast with the behaviour of the LiNi_0.5_Mn_0.5_O_2_ electrode. The potential profiles show that the initial discharge capacity located in the range 230–260 mAh·g^−1^ is retained at 200–220 mAh·g^−1^ after 30 cycles, while the stoichiometric compound LiNi_0.5_Mn_0.5_O_2_ only displays an initial discharge capacity of 180 mAh·g^−1^.

The charge–discharge reactions of a Li-rich electrode are described by the activation of the two components LiNi_0.5_Mn_0.5_O_2_ and Li_2_MnO_3_ as follows [51]. Let us consider the case of Li_1.2_Ni_0.2_Mn_0.6_O_2_, otherwise written as 0.5Li_2_MnO_3_•0.5LiNi_0.5_Mn_0.5_O_2_. 

(1) First step of charge reaction by activation of the $R\bar{3}m$ phase with a regular Li^+^ extraction from the 3*b* site of the layered lattice:

0.5Li_2_MnO_3_•0.5LiNi_0.5_Mn_0.5_O_2_ → 0.5Li_2_MnO_3_•0.5Li_0.5_Ni_0.5_Mn_0.5_O_2_ + 0.5Li^+^ + 0.5e^−^. (8)

(2) Second step of charge reaction by activation of Li_2_MnO_3_ inducing loss of Li and oxygen release at the end of the charge i.e., potential above 4.5 V:

0.5Li_2_MnO_3_•0.5Li_0.5_Ni_0.5_Mn_0.5_O_2_ → (0.5 − *α*)Li_2_MnO_3_•*α*MnO_2_•0.5Li_0.5_Ni_0.5_Mn_0.5_O_2_ + 2*α*Li^+^ + 2*α*e^−^ + 0.5*α*O_2_. (9)

(3) Discharge process by the insertion of the $R\bar{3}m$ phase and formation of the new LiMnO_2_ lithiated phase:

(0.5 − *α*)Li_2_MnO_3_•*α*MnO_2_•0.5Li_0.5_Ni_0.5_Mn_0.5_O_2_ + (0.5 + *α*)Li^+^ + (0.5 + *α*)e^−^ → (0.5 − *α*)Li_2_MnO_3_•*α*LiMnO_2_•0.5LiNi_0.5_Mn_0.5_O_2_. (10)

**Figure 22:**
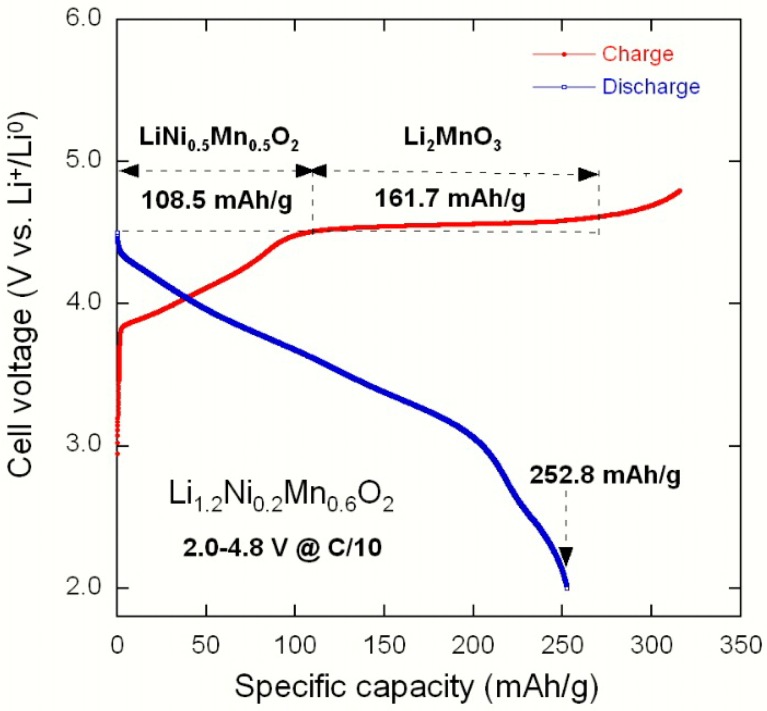
The first charge–discharge profile of a Li//Li_1.2_Ni_0.2_Mn_0.6_O_2_ cell cycled at C/10, showing the mechanism of the activation of Li_2_MnO_3_ and LiNi_0.5_Mn_0.5_O_2_ components.

**Figure 23:**
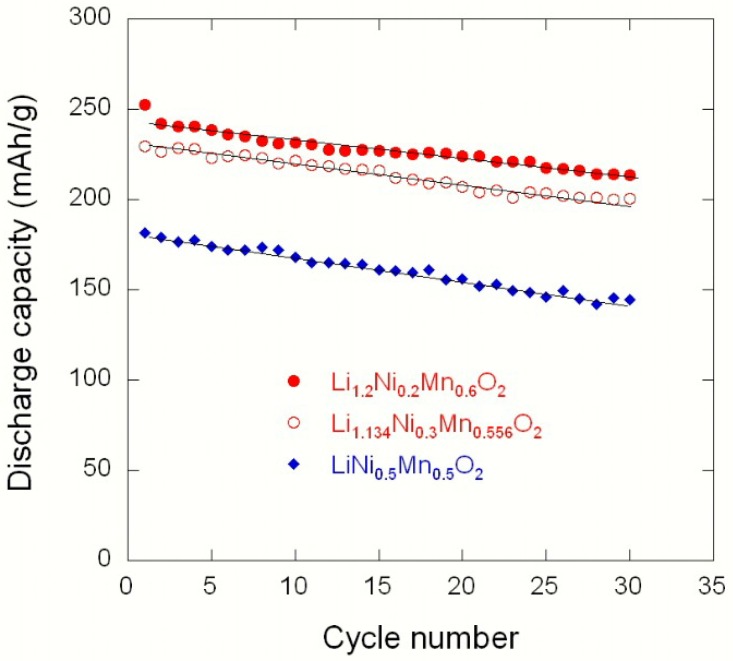
Cyclic performance of Li//Li(Ni_x_Li_(1/3-2x/3)_Mn_(2/3-x/3)_)O_2_ electrodes at C/10.

Figure 22 illustrates the first charge–discharge profile of a Li//Li_1.2_Ni_0.2_Mn_0.6_O_2_ cell cycled at C/10, showing the mechanism of the activation of Li_2_MnO_3_ and LiNi_0.5_Mn_0.5_O_2_ components. The typical features of the Li//Li(Ni_x_Li_(1/3-2x/3)_Mn_(2/3-x/3)_)O_2_ coin cells cycled at C/10 in the voltage range 2.0–4.8 V vs. Li^0^/Li^+^ are shown in Figure 23. With an initial discharge capacity of 252.8 mAh·g^−1^, this electrode, Li_1.2_Ni_0.2_Mn_0.6_O_2_, appears to be the best among the studied materials. The capacity decreased slightly upon cycling at the rate of 1.01 mAh·g^−1^ per cycle. These results are comparable to those in the literature [52,53,54]. Liu et al. [53] reported a discharge capacity 253 mAh·g^−1^ for MnO_2_-coated Li_1.2_Ni_0.2_Mn_0.6_O_2_ cycled at C/10. Zhao et al. [54] showed that the same electrode materials doped with Sn delivered a lower initial discharge capacity of 212.6 mAh·g^−1^, which seems to be due to the formation of big secondary particles synthesized via a carbonate co-precipitation method.

According to Figure 23, we find here that the Ni doping has improved the capacity but it had no effect on the cycling stability, since the slope of the capacity as a function of the cycle number is the same for the different compositions. This is in contrast with prior conclusions of Lu et al., who found that the cycling stability was significantly enhanced in samples with *x* > 1/4 in Li(Ni_x_Li_1/3-2x/3_Mn_2/3-x/3_)O_2_ [55,56,57]. Indeed, the stability of the Li-rich materials is the main problem that still has to be solved before any application can be envisioned [58,59,60,61,62]. The characterization of a 0.5Li_2_MnO_3_·0.5LiMn_0.42_Ni_0.42_Co_0.16_O_2_ “composite” by the synchrotron X-ray powder diffraction has shown complex reaction pathways that depend on the current density [63]. As the Li content relative to Ni and Mn decreased, the LLL materials were observed to be either single-phase layered, two-phase layered-rocksalt, or three-phase layered-rocksalt-spinel, depending on their location within the Li-Mn-Ni-O phase diagram [64]. Gu et al. have shown that, upon 300 cycles of charge/discharge, the structure of Li_1.2_Ni_0.2_Mn_0.6_O_2_ is transformed into a spinel structure [65]. Therefore, the evolution of the LLLs and their related degradation upon cycling is a complex problem that is not entirely understood—in contrast with the NMC case, where the delithiation proceeds in two well-identified steps in Li_x_Ni_y_Mn_y_Co_1−2y_O_2_: the first step of the delithiation is associated with the redox reaction involving the nickel ions: Ni^2+^ → Ni^3+^ → Ni^4+^ in the composition range 1 > *x >* 1 − 2*y*; the second step is the redox process Co^3+^ → Co^4+^ in the range *x* < 1 − 2*y*, as there is practically no overlap between the two redox reactions [38].

## 4. Concluding Remarks

In this work we have examined several cathode materials for Li-ion batteries, all of them crystallizing in a layered network. The effect of the synthesis procedure on the cationic distribution in NMC compounds with various configurations and the merit of the hierarchical nano-/micro-structures was investigated. The local Ni^2+^ arrangement on the lithium layers has been estimated as a function of the composition, the Li/metal ratio, and the metal/chelating agent used in the sample preparation. It has been demonstrated that the high crystallinity is essential to obtain accurate electrochemical performance and to maintain the structural integrity of the electrode lattice during cycling. Among the mixed transition metal oxides, Li_1/3_Ni_1/3_Mn_1/3_CoO_2_ appears to be an attractive material because the combination of Ni, Mn, and Co can provide many advantages such as high specific capacity compared with LiCoO_2_. Its good structural integrity is due to the absence of Mn^3+^ Jahn–Teller ions. Both Rietveld refinements and magnetic measurements have shown that the Li/Ni cation mixing on the 3*b* Wyckoff site of the interslab space was very small (concentration of Ni^2+^-3*b* ions lower than 2%) and consistent with the structural model (Li_1-*δ*_Ni*_δ_*)_3*b*_(Li*_δ_*Ni_x-*δ*_Mn_y_Co_1-x-y_)_3*a*_O_2_ results obtained by adjusting parameters of synthesis. Electrochemical tests carried out by galvanostatic charge–discharge cycling reflected the high degree of sample optimization.

A significant increase in the specific capacity was obtained with Li_2_MnO_3_ and LiNi_0.5_Mn_0.5_O_2_ composites (Li(Ni_x_Li_(1/3-2x/3)_Mn_(2/3-x/3)_)O_2_ with 0.0 ≤ *x* ≤ 0.5), which are Li-rich compounds having an intergrown structure (or integrated layered-layered). Their specific capacity reached 200 mAh·g^−1^ after 30 cycles or more. However, while the mechanism of the lithiation–delithiation process is well understood in NMC materials, which in addition is reversible, that of the Li-rich compounds is still under debate and the problem of degradation upon cycling is not yet solved. The rate capability is a problem that seems more difficult to overcome. Li_1.2_Ni_0.2_Mn_0.6_O_2_ core encapsulated by a nanospinel (LiNi_x_Mn_2-x_O_4_) layer with the thickness of about 10 nm combined the advantages of the highly conductive spinel surface and high capacity layered core was able to deliver 274.6 mAh·g^−1^ at 1C rate [61]. Song et al. used graphene oxide (GO) to wrap their Li(Li_0.2_Mn_0.54_Ni_0.13_Co_0.13_)O_2_ material [66]. In addition to the highly conductive cubic spinel transformed from the layered phase on the surface during electrochemical cycling [67], the graphene oxide was also reduced. As a result, this composite delivered 201 mAh·g^−1^ at a current density of 2500 mA·g^−1^. However, even if coating the LLL particles with different materials improves the capacity retention, none of them is currently able to prevent the degradation upon many cycles. This is thus the main problem that remains to be solved, together with the voltage decay of LLLs over a long period of cycling, before these promising materials can find an industrial development.

## Figures and Tables

**Table 1:** Lattice parameters *a*, *c*, *c*/*a* of NMC materials, synthesized by solid-state reaction. *V* is the volume of the unit cell. *L* is the mean lattice coherence length. The last column is the ratio of the intensities of the XRD lines labelled by their Miller indexes and *R* = (*I*_006_ + *I*_102_)/*I*_101_. The parameter *η* reveals the site-exchange rate of Ni^2+^(3*b*) cation mixing.

Sample	*a* (Å)	*c* (Å)	*c*/*a*	*V* (Å^3^)	*L* (nm)	*I*_003_/*I*_104_	*R*	*η* (%)
550	2.8833(2)	14.3251(2)	4.948	119.08(9)	59.9	1.197	0.420	6.70
4_½_4_½_1	2.8766(8)	14.3005(5)	4.971	118.33(2)	65.5	1.209	0.418	5.31
532	2.8732(2)	14.2684(4)	4.966	102.01(2)	61.6	1.252	0.415	3.34
442	2.8685(1)	14.2655(3)	4.973	101.65(1)	64.6	1.425	0.401	2.97
333	2.8604(2)	14.2376(2)	4.977	100.88(2)	72.3	1.456	0.387	1.56
334	2.8622(3)	14.2455(7)	4.977	116.70(2)	63.8	1.450	0.395	1.77
¼¼½	2.8628(5)	14.2259(6)	4.981	116.86(6)	62.0	1.452	0.390	0.70

**Table 2:** Electrochemical parameters of NMC materials calculated from Equation (4) at low rate of C/20. (1 − 2y) is the cobalt concentration in the LiNi_y_Mn_y_Co_1-2y_O_2_ structure.

Cathode	(1 − 2y)	*E*_0,1_ (V)	−*K*_1_	*E*_0,2_ (V)	−K**_2_
550	0.00	3.737	3.82	3.869	3.12
4_½_4_½_1	0.10	3.734	3.75	3.903	2.98
442	0.20	3.731	3.75	3.955	2.98
333	0.33	3.722	3.71	3.932	3.00
334	0.40	3.717	3.78	3.921	3.01

**Table 3:** Crystallographic parameter of Li_1+x_(NMC)_1-x_O_2_ materials. *η*_R_ and *η*_M_ reveal the site-exchange rate of Ni^2+^(3*b*) cation mixing estimated from Rietveld refinements and magnetometry, respectively.

(1 + *x*)	*a* (Å)	*c* (Å)	*c*/*a*	*V* (Å^3^)	*L* (nm)	*η*_R_ (%)	*η*_M_ (%)
1.02	2.8650(1)	14.2252(2)	4.975	101.31(2)	59.4	1.98	1.88
1.04	2.8603(2)	14.241(2)	4.979	100.90(3)	55.9	1.43	1.50

**Table 4:** Structural data of the LiNi_1/3_Mn_1/3_Co_1/3_O_2_ samples synthesized at different values of *R*. *a*, *c* are the lattice parameters, *V* is the volume of the unit cell, and *L* the coherence length (crystallite size). The coherence length is the arithmetic average of the length given by the Scherrer law applied to three main XRD lines. The two next lines are relative intensities of the XRD lines that provide insight on the concentration of Ni^2+^(3*b*) defects. The other X-ray Rietveld refinement parameters are reported in the last lines. *S*(*M*O_2_) = 2(⅓-*z*_oxy_)*c* is the thickness of the metal–O_2_ planes, *I*(LiO_2_) = *c*/3-*S*(*M*O_2_) is the thickness of the interslab space; other notations are conventional.

Crystal Data	*R* = 0.5	*R* = 1.0	*R* = 2.0	*R* = 3.0	*R* = 4.0
*a* (Å)	2.8680(2)	2.8671(2)	2.8602(1)	2.8675(2)	2.8684(3)
*c* (Å)	14.2588(3)	14.2593(2)	14.2458(2)	14.2589(4)	14.2619(3)
*c*/*a*	4.9716(4)	4.9734(4)	4.9807(2)	4.9726(5)	4.9720(6)
*V* (Å ^3^)	101.57(2)	101.51(2)	100.92(1)	101.53(2)	101.62(2)
*L* (Å)	≈570	≈540	≈490	≈550	≈610
*I*_(003)_/*I*_(104)_	1.440	1.614	1.728	1.558	1.423
(*I*_(006)_ + *I*_(102)_)/*I*_(101)_	0.482	0.427	0.378	0.438	0.459
*S*(*M*O_2_) (Å)	2.126(2)	2.120(5)	2.109(3)	2.123(3)	2.126(2)
*I*(LiO_2_) (Å)	2.627(2)	2.633(6)	2.640(3)	2.630(3)	2.628(3)
*η* (%)	2.96	2.04	1.26	2.90	3.0
